# Looking at paintings in the Vincent Van Gogh Museum: Eye movement patterns of children and adults

**DOI:** 10.1371/journal.pone.0178912

**Published:** 2017-06-21

**Authors:** Francesco Walker, Berno Bucker, Nicola C. Anderson, Daniel Schreij, Jan Theeuwes

**Affiliations:** Department of Experimental and Applied Psychology, Vrije Universiteit Amsterdam, Amsterdam, The Netherlands; State University of New York Downstate Medical Center, UNITED STATES

## Abstract

In the present study, we examined the eye movement behaviour of children and adults looking at five Van Gogh paintings in the Van Gogh Museum, Amsterdam. The goal of the study was to determine the role of top-down and bottom-up attentional processes in the first stages of participants’ aesthetic experience. Bottom-up processes were quantified by determining a salience map for each painting. Top-down processing was manipulated by first allowing participants to view the paintings freely, then providing background information about each painting, and then allowing them to view the paintings a second time. The salience analysis showed differences between the eye movement behaviour of children and adults, and differences between the two phases. In the children, the first five fixations during the free viewing phase were strongly related to visually salient features of the paintings—indicating a strong role for bottom-up factors. In the second phase, after children had received background information, top-down factors played a more prominent role. By contrast, adults’ observed patterns were similar in both phases, indicating that bottom-up processes did not play a major role when they viewed the paintings. In the second phase, children and adults both spent more time looking at regions that were mentioned in the background information. This effect was greater for adults than for children, confirming the notion that adults, when viewing paintings, rely much more on top-down processing than children.

## Introduction

Even though more and more paintings are available online, an increasing number of people prefer to visit museums to experience and appreciate the original artworks. While appreciating paintings, we make multiple eye movements, which may reveal the complex cognitive and perceptual components underlying our aesthetic experience (e.g., [1]).

A lot is known about eye movements and art perception in experimental settings. In a classic study, Yarbus analyzed the eye movement pattern of an observer viewing "They Did Not Expect Him", a painting by Répin. Using a suction cup, Yarbus tracked the observers’ eyes. The study revealed that observers focus on different areas of the painting (e.g., people, objects) depending on task instructions and context [2]. In line with these findings, other studies show that participants' eye movement patterns can be influenced by expertise [3], culture [4], personality traits [5] and the physical salience of features in the painting [6, 7].

It is generally believed that eye movements are the result of the visual (bottom-up) input reaching the eye and the expectations and intentions of the observer (top-down goals) [8]. Bottom-up attentional processes are stimulus driven and automatic, directly determined by the physical properties of the environment; while top-down attentional processes are determined by goals, intentions and interpretation of the observer [9]. Nevertheless, even when people have a clear top-down goal, a bright, yet irrelevant object may still capture their attention [10, 11]. It is this interplay between input from the outside world and the goals and intentions of the observer that ultimately determines where people look, where they look next and how long they remain fixated at a particular location. When viewing pictures, an observer can quickly extract the gist of the scene (i.e., what the picture is about), but details of objects and appreciation of the picture become available only after subsequent scanning of the image.

Models that stress the role of low level (bottom-up) factors propose that the eyes are mainly driven toward regions in a picture that are physically salient. The algorithm developed by Itti and Koch ([12], see also [13]) generates a salience map, showing the visual salience of different parts of an image on the basis of its physical properties (in particular, peaks in the distribution of brightness, color and orientation). According to bottom-up models of attention, salient regions are inspected before others. For example, Underwood and Foulsham [14] recorded fixations while viewers inspected pictures of room interiors that contained objects with known salience characteristics. In this setting, highly salient objects attracted earlier fixations than less conspicuous objects, but only when viewers were asked to inspect and encode a whole picture. If viewers had a specific instruction, i.e., a strong top-down goal (e.g., when they had to find a small target within the scene), the salience of the objects did not predict the order of fixations. Similar findings have been reported in Parkhurst Law and Niebur [15], in a study where viewers looked at photographs of home interiors, buildings, city scenes, natural environments, as well as computer-generated fractals. Here, the salience values of the different regions were a good predictor of the order of fixations for all categories of photographs, but only when participants had no particular task instruction other than “look around at the images”. In other words, bottom-up factors can accurately predict eye movement patterns if an observer has no strong top-down search goal or strategy.

If people have a strong top-down goal and/or particular expectations, the salience of the regions plays a much smaller role in determining eye movements. When participants perform a task in a natural environment, their eyes fixate regions that are particularly relevant to the task [16]. Observations of participants performing natural tasks such as driving, walking, sports, and making tea or sandwiches yield similar results (e.g., [17, 18]). Crucially, participants performing these natural tasks seldom fixate areas that are irrelevant for the task at hand: Each fixation is related to one specific component of the task (for example, looking at the water spout when filling a kettle).

Studies investigating visual aesthetics have also found evidence that top-down factors play a role. For example, authors have shown that task requirements (i.e., no instruction, versus instructions to remember content features, versus instructions to concentrate on specific artistic aspects of the artwork) have a large effect on eye movement patterns [19]. Others have demonstrated that content related top-down processing prevails over low-level bottom-up processing, especially when a painting includes a human subject [20].

Museum professionals believe that the museum setting has a strong positive influence on visitors’ perception and evaluation of the paintings displayed [21]. Nevertheless, most studies that have investigated the eye movement patterns of participants looking at paintings have been conducted in the laboratory, using pictures on a computer screen. Even if this method provides full control over properties of the picture (e.g., size, color, light, etc.), the task of the viewer, the eye tracking methodology and other confounders, there is evidence that participants’ experience is very different from that of a visit to the museum. Several studies show, in fact, that context can strongly influence the overall aesthetic experience (e.g., [22, 23, 24, 25]). Smith and Smith [22]—the first to empirically investigate the time visitors spent viewing artworks in a real museum setting—showed that museum visitors viewed each of six selected artworks for an average time of 27.2 seconds [22]. This is remarkable since viewing times in classical laboratory settings are typically less than 3 seconds [23]. This result was recently replicated and validated in a new study by Smith and Smith [24] and in a study by Carbon [23]. Another recent study by Brieber and colleagues [25] directly compared participants’ aesthetic experience in a laboratory and a museum setting. In this study, participants were divided into two groups, based on the context in which the art exhibition was viewed: One group viewed an exhibition in an actual museum, while the other group viewed a replica of the exhibition, presented on screen in a laboratory setting [25]. Brieber et al.’s [25] results not only confirmed differences between the two groups in the time spent viewing the paintings, but also showed that participants liked the artworks more and found them more interesting in the museum setting [25]. Another study conducted by the same group showed that participants found artworks more arousing and positive in the museum than in the laboratory [26]. The study also showed that the museum context enhanced memory formation: Participants who viewed the exhibition in the museum were better able to recall the artworks than participants in the laboratory [26]. Other studies analyzing participants’ reactions to paintings displayed in a museum, compared to other forms of display (i.e., on paper, on a computer screen, projection on a larger screen), provide confirming evidence that context can strongly modulate the overall aesthetic experience [27, 28, 29].

These previous studies suggest that ecologically valid testing in museum conditions is essential for empirical aesthetics. One way of achieving this is through the use of mobile eye-tracking technology. A number of studies have already used the technology in this way. Quian Quiroga et al. [30] asked six participants to view Millais’ Ophelia, displayed at the Tate Britain. Another group of eight participants was asked to view the same painting in a laboratory setting. The authors showed that, in the laboratory, participants’ fixations were mostly concentrated around the figure of Ophelia, while museum participants tended to focus less on Ophelia, and more on the undergrowth painted behind the female figure [30]. In a recent study by Heidenreich and Turano [31], the authors asked four participants to view fourteen artworks displayed in a museum. Their results suggest that eye movement patterns change over time. Furthermore, no correlation was found between viewing time and art appreciation [31]. This latter result is inconsistent with what shown in [25]. However, Brieber and colleagues [25] have argued that Heidenreich and Turano’s [31] study is limited by small sample size (i.e., *N* = 4). The same limitation applies to the study by Quian Quiroga [30], in which only six participants were assigned to the “museum condition”. By contrast, the study by Brieber et al. [25] had 22 participants in the “museum condition”.

In the present study, mobile eye-tracking technology was used to investigate what factors affected the eye movement patterns of observers viewing actual paintings on display at the Van Gogh Museum, Amsterdam. More precisely, we were interested in the influence of bottom-up and top-down attentional processes on participants’ gaze behaviour–a question that has never been tackled in this way in an actual museum setting. It is well known that art is most appreciated by those who are well-informed about the painting, the painter and the relevant background [32, 33, 34]. To test the role of such top-down factors, we introduced two conditions: In a first phase, we examined visitors’ spontaneous eye movement behaviour, and in a subsequent second phase we examined their eye movement behaviour after they had received specific information about each painting.

To further investigate the role of top-down and bottom-up factors, we compared “eye movements in natural behaviour” [35] of adults and primary school children. In a recent eye movement study it was shown that with age, bottom-up fixation selection becomes weaker as top-down factors affecting eye movement selection become more important [36]. Furthermore, it is known that the efficiency of bottom-up processing declines in later adulthood [37, 38, 39], while top-down attentional guidance is strongly preserved [37]. By comparing eye movement patterns between children and adults, we thus indirectly manipulated the extent to which bottom-up and top-down factors determine eye movement patterns. To our knowledge, this study is the first to compare the eye movement behaviour of children and adults in an actual museum setting.

## Method

### Participants & stimuli

The experiment took place in the Van Gogh Museum in Amsterdam on four separate days during regular visiting hours. Twelve children (6 female, age between 11–12, mean = 11.17, SD = 0.37) were tested on two weekdays between 9:00 and 14:00. Twelve adults (6 female, age between 20–29, mean = 23.25, SD = 2.68) were tested on two other weekdays between 17:00 and 21:30. The children were recruited from a primary school (the Woutertje Pieterse Basis School, Leiden, The Netherlands) and the adults were recruited from the Vrije Universiteit Amsterdam. All participants reported that they had normal vision. None wore glasses or contact lenses. The participants had not attended art school at any level and had never visited the Van Gogh Museum, Amsterdam. All adult participants gave written informed consent before participation. An ad-hoc informed consent form was developed for the children and was signed by the children's parents prior to participation. All participants received a free entry ticket to the Van Gogh Museum and adults were paid an additional 5 euro. The study was approved by the ethics board of the Faculty of Psychology and Education and conducted according to the principles of the Declaration of Helsinki.

**Fig 1:**
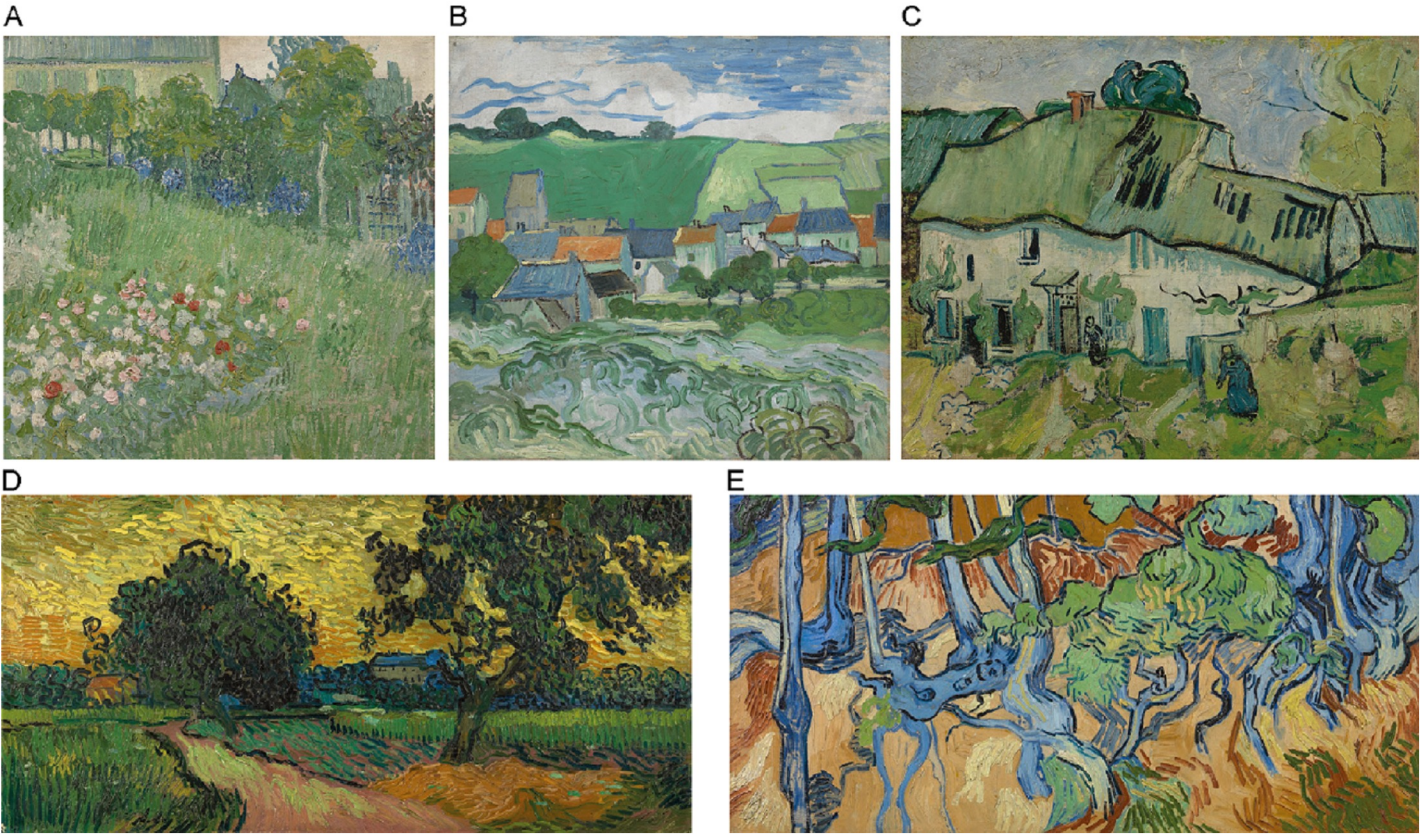
The five art paintings of Van Gogh selected as stimulus material. A) Daubigny’s Garden (1890). B) View of Auvers (1890). C) Farmhouse (1890). D) Landscape at Twilight (1890). E) Tree Roots (1890). Painting images downloaded from the official website of the Van Gogh Museum, Amsterdam, under a CC BY license, with permission from J. van Kregten, Van Gogh Museum, Amsterdam (Vincent van Gogh Foundation).

Five art paintings on display in the same room of the museum were selected as stimulus material. These paintings were selected because they were displayed in the same room and were not particularly famous, making it unlikely that the participants had seen the paintings before. The paintings were assigned sequence numbers: (1) Daubigny’s Garden, (2) View of Auvers, (3) Farmhouse, (4) Landscape at Twilight and (5) Tree Roots. The order in which the paintings were viewed for the experiment was counterbalanced between participants; half of the children and half of the adults viewed the paintings from one to five, while the others viewed the paintings from five to one. All paintings were viewed at a distance of approximately 3 meters. The paintings had the following dimensions (height x width) in centimeters: Daubigny’s Garden (51.0 x 51.2), View of Auvers (50.2 x 52.5), Farmhouse (38.9 x 46.4), Landscape at Twilight (50.2 x 101.0) and Tree Roots (50.3 x 100.1). All the paintings were painted by Vincent Van Gogh in the year 1890 (see Fig 1).

### Apparatus

Monocular eye movements of the right eye were tracked with a head-mounted mobile eye-tracker, developed by Pupil Labs [40]. The eye-tracker weighed 28 grams and consisted of an eye camera, to track movements of the eye, and a world camera, to record where participants were looking (i.e., the external world). The eye camera was an infrared camera with infrared illumination for dark pupil tracking, and had a resolution of 640 x 480 at 30 frames per second. The world camera had an auto focus lens with a 90 degrees diagonal field of view, and a resolution of 1910 x 1080 pixels at 30 frames per second. The mobile eye-tracker was connected to a Toshiba Portege Z30-A-12X laptop, running the Ubuntu 15.04 operating system and Pupil software [41] to record eye movements and the external world. Before the mobile eye-tracker was adjusted on their head, participants put on a backpack. After the calibration procedure, the laptop was carefully placed into the backpack and participants were asked not to touch the eye-tracker. Participants were asked to look and move around naturally, but to avoid brusque head movements.

### Procedure

At the start of the experiment, participants were welcomed in the calibration room and put on the backpack and the head-mounted eye-tracker. To calibrate the eye-tracker, participants were placed three meters in front of a wall on which 12 small markers were positioned in a (4 x 3, height x width) grid covering the largest part of the field of view. Participants were instructed to focus on the specific markers in the visual scene, and the Pupil software was used to calibrate the eye-tracker (see Natural Features Calibration, [40]). After calibration, participants were given instructions about what they should do when they arrived in the room where the paintings were displayed.

The experiment took place in two phases. In both phases, participants were instructed to look for 30 seconds at each painting. The experimenters made sure that each painting was viewed for exactly 30 seconds, that the distance between the participant and the paintings was always approximately 3 meters, and that other visitors did not block the view of the paintings. In phase 1, participants were instructed to view each painting freely (i.e., they were not given a particular task nor asked to look at specific features of the paintings). In phase 2, they were read a short description before viewing each painting. Participants could not see the paintings while the descriptions were being read. At the end of phase 1 and phase 2, participants were guided to a separate room for a short post phase interview. During the interview, an image of each painting was presented to the participants, after which they were asked to respond to the open-ended question "What struck you about this painting?”. Responses were recorded with a voice recorder and later transcribed for analysis. Since the experiment was conducted during the regular opening hours of the museum (opening the museum afterhours was not possible for security reasons), there were some uncontrolled differences between the test conditions for different participants.

### Painting descriptions

The painting descriptions were developed by the educational staff of the Van Gogh Museum, Amsterdam. Descriptions for adults were in English. Descriptions for children were translated into Dutch by a professional translator belonging to the museum staff. The English and Dutch descriptions were kept as simple as possible, focusing on the same features of the paintings (see Appendix).

### Analyses

To mark the paintings, four ~10 cm x ~10 cm paper markers were placed on the wall, near the corners of each painting. These allowed the Pupil software to digitally extract the surfaces of the paintings. Eye movements that landed outside the surfaces of the paintings were excluded from the analysis.

#### Salience maps and baseline-corrected salience values

**Fig 2:**
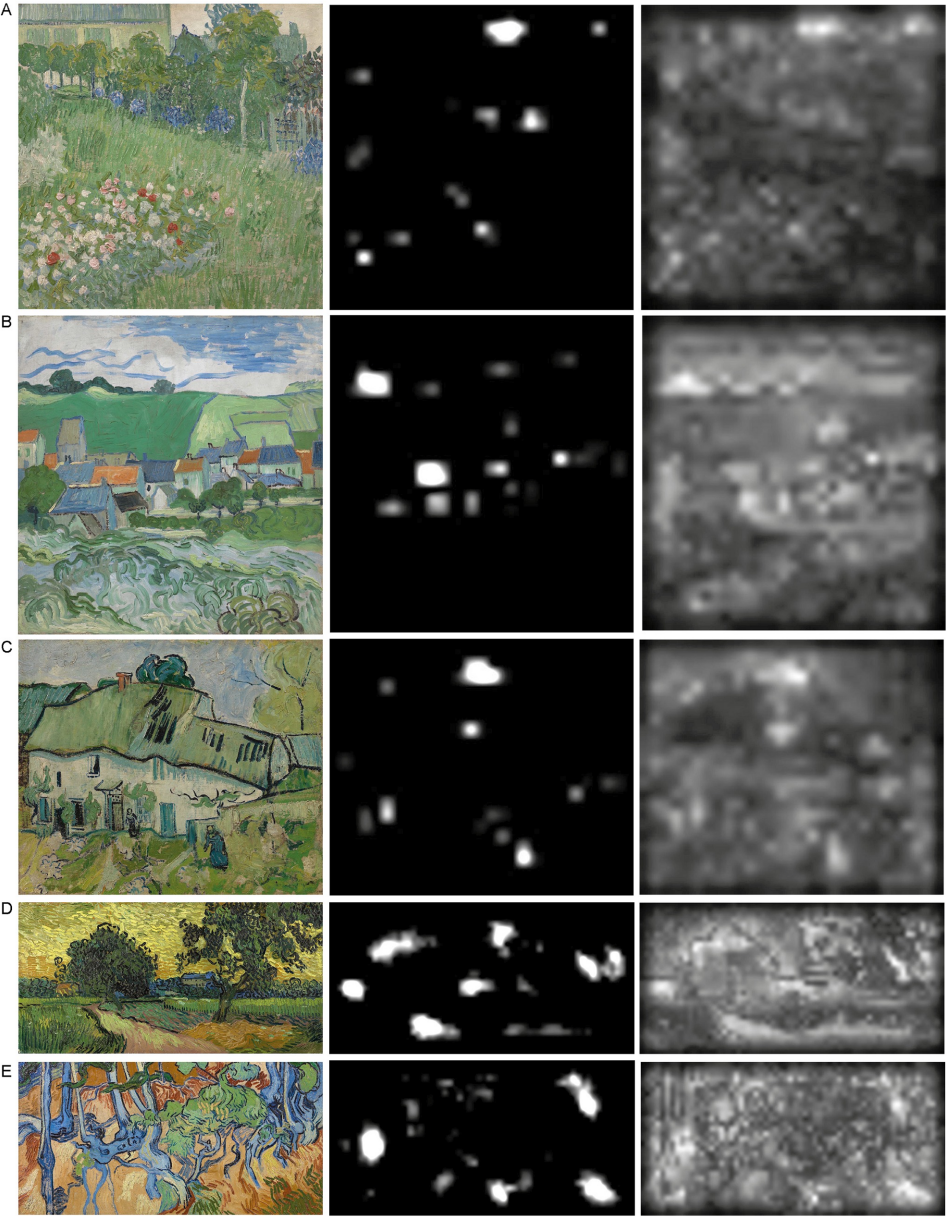
Salience maps. The left panels show the original paintings, ordered as in Fig 1. The middle panels show the salience maps, computed by the Salience Toolbox [13]. The right panels show the final salience maps, used for our analysis; these do not include any inhibitory processes, and are thus smother compared to the maps presented in the middle panels. Painting images downloaded from the official website of the Van Gogh Museum, Amsterdam, under a CC BY license, with permission from J. van Kregten, Van Gogh Museum, Amsterdam (Vincent van Gogh Foundation).

To assess the influence of bottom-up factors, feature-based image statistics on eye movement behaviour, salience maps for each image were calculated using the Salience Toolbox [13]. This toolbox analyzes each painting in terms of its low-level intensity, color and orientation channels and produces a map showing regions that stand out from their surroundings in terms of these features. Bright regions on the map represent areas that are relatively salient (see Fig 2). To produce the maps, we used an intermediate stage in the output from the salience modeling procedure that does not include any inhibitory processes. This made it possible to generate a smoother map than would otherwise have been possible, and to increase the variability of salience values across the map (see [42]). The resulting salience maps were normalized to values between 0 and 100.

A common way to determine how the salience model predicts fixation locations is to compare the places that participants looked at in the paintings to the values produced by the salience model at the same locations. This measure is often termed “salience at fixation” (see [42]). Here, salience at fixation was defined as the average of the salience map values within a 40 x 40 pixel region (approximately 0.75 x 0.75 degrees visual angle) around each of a participant’s first five fixation locations. To determine whether low-level image features influence fixation location over and above what might be expected by chance, salience at fixation was compared to a baseline level. This baseline is often calculated by creating a random data set; for example, by generating random fixation locations distributed evenly across an image and then calculating the salience at fixation at those locations. Here, we chose a conservative baseline value that takes into account any spatial or sequential biases inherent in fixation data. For example, fixations early on in scene viewing tend to land somewhere in the middle of the picture [43]. Previous work has also shown that salience at fixation tends to be higher for earlier fixations [15]. To account for these regularities, we calculated a baseline salience at fixation value for each fixation number for each participant. For example, the baseline for salience at fixation for the 1^st^ fixation was calculated by averaging the salience at fixation values for all of the 1^st^ fixations that the participant made over each painting. That is, if a participant made their first saccade at an x,y location of (400, 300) in the first painting, then salience at fixation was calculated around this location in all of the paintings. Because the paintings were of different sizes, this location was interpolated to the location commensurate with the size and shape of the different image. These values were then averaged to give the participant’s baseline value for the first fixation. This was done for each fixation number. Thus, the baseline values represent a conservative salience at fixation value that might be expected based on the fixation number and the general idiosyncratic tendencies of the participant. These baseline values were then subtracted from the raw salience values at fixation for fixations one to five. Thus, values above 0 represent higher than expected salience at fixation, and values below 0 represent lower than expected salience at fixation (see [44]). These baseline values were calculated separately per phase. Since visual salience is known to play a prominent role in guiding eye movements during the first five fixations (e.g., [45]), we restricted our analysis to these fixations.

#### Regions of Interest

**Fig 3:**
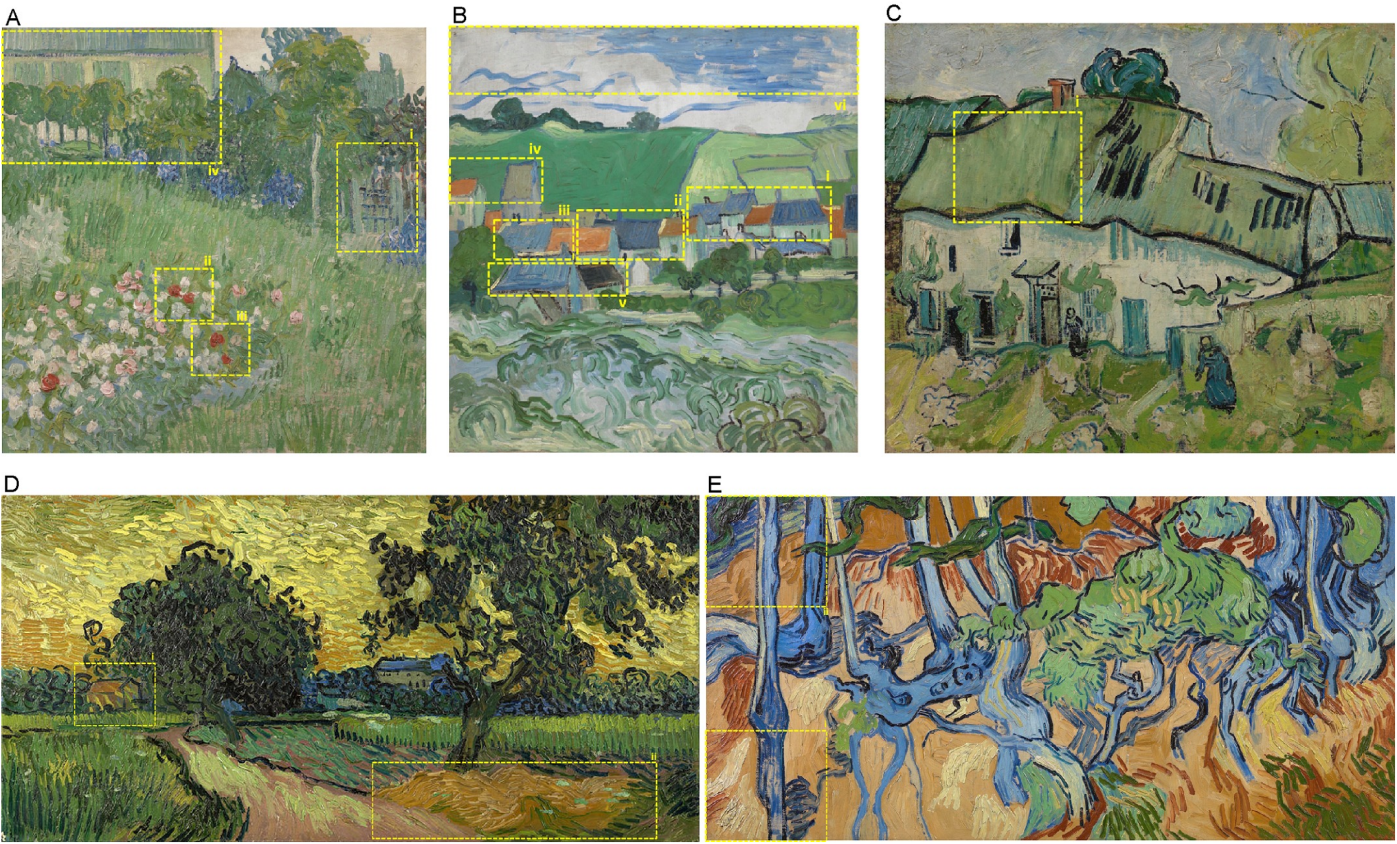
Regions of Interest (dashed yellow lines) for the five selected paintings. Daubigny’s Garden (A) contained four ROI’s; View of Auvers (B) contained six ROI’s; Farmhouse (C) contained one ROI; Landscape at Twilight (D) contained two ROI’s; Tree Roots (E) contained two ROI’s. Painting images downloaded from the official website of the Van Gogh Museum, Amsterdam, under a CC BY license, with permission from J. van Kregten, Van Gogh Museum, Amsterdam (Vincent van Gogh Foundation).

To examine whether eye movements were influenced by the information participants received during phase 2, we defined Regions of Interest (ROI’s) for each painting, based on the painting descriptions given to participants. Daubigny’s Garden contained four ROI’s, (i) the gate, (ii) the roses at the top, (iii) the roses at the bottom, and (iv) the house. View of Auvers contained six ROI’s, (i) the roofs at the right, (ii) the roofs at the center, (iii) the roofs at the left, (iv) the roofs at the top, (v) the roofs at the bottom, and (vi) the sky. Farmhouse contained one ROI, (i) part of the roof of the house. Landscape at Twilight contained two ROI’s, (i) the house on the left, and (ii) the hay. Tree Roots contained two predefined ROI’s, (i) the lower left root, and (ii) the upper left sky. All ROI’s used for the ROI analysis are displayed in Fig 3. Based on the participants’ descriptions during the post phase interviews, we also defined one additional ROI, a “human figure”, in the painting Tree Roots. This new ROI was used for a separate analysis.

To investigate the influence of the descriptions that were read to the participants between phases 1 and 2, we calculated the fixation time spent in the ROI’s as a percentage of the total fixation time spent on the painting surface. To do so, we multiplied all fixations that fell inside the painting surface by their fixation duration, and classified them as falling inside or outside the ROI’s of that particular painting. Then, the total fixation duration inside the ROI’s was divided by the total fixation duration spent on the painting surface to get the percentage of time spent in the ROI’s.

We went on to calculate the percentage of ROI’s reported by each participant in each phase. For the interview analysis we collapsed the two ROI’s containing “roses” in the painting Daubigny’s Garden, into a single ROI. For the painting View of Auvers we collapsed the five ROI’s containing “roof tops”, to make just two ROI’s (roof tops and sky). For all other paintings, the ROI’s remained unchanged, with Farmhouse having one ROI (part of the roof), Landscape at Twilight having two (house on the left and hay) and Tree Roots having two (lower left root and upper left sky).

To visualize viewing patterns, we created separate heat maps for each painting and for each phase. Separate heat maps were generated for adults and for children. All fixations were weighted by duration and plotted on the painting surface (800 x 800 grid) using a Gaussian outflow filter (Kernel standard deviation = 25).

### Exclusions

Participants with less than 5 fixations on one or more paintings during phase 1 or phase 2 were excluded from the analysis. Three children, but no adults, were excluded on these grounds.

### Data

The data is freely available at the following link: http://doi.org/10.17605/OSF.IO/8MX7U.

## Results

### Description

Considering all fixations recorded during the study, each adult made an average of 63 fixations on the surfaces of the paintings (88.3% of total fixations) (see Table 1). Each child made an average of 53 fixations (78.1%) (see Table 2). All other fixations fell outside the surface of the paintings.

**Table 1:** Adults’ mean number of fixations on surface and mean percentage of total fixations on surface. Note that the names of the paintings are abbreviated.

Adults (*N* = 12)	Daubigny	Auvers	Farmhouse	Twilight	Tree Roots
Phase1					
Number	62	63.8	55	57	66
Percentage	87.7%	88.1%	84.0%	90.8%	95.6%
Phase2					
Number	64	69	62	65	67
Percentage	92.5%	71.2%	83.9%	94.7%	94.1%

**Table 2:** Children’s mean number of fixations on surface and mean percentage of total fixations on surface. Note that the names of the paintings are abbreviated.

Children (*N* = 9)	Daubigny	Auvers	Farmhouse	Twilight	Tree Roots
Phase1					
Number	49	52	53	48	52
Percentage	80.8%	71.5%	60.9%	91.1%	83.8%
Phase2					
Number	59	56	57	52	55
Percentage	88.0%	77.1%	66.4%	80.6%	80. 7%

### Salience at fixation

An analysis of variance (ANOVA) on mean baseline-corrected salience at fixation with painting (painting 1–5), fixation index (fixation 1–5), and phase (phase 1/phase 2) as within-subject factors and group (children/adults) as a between-subjects factor, showed a significant main effect of painting, *F*(4, 76) = 48.450, *p <* .001, η_p_^2^ = .718, a marginally significant main effect of phase, *F*(1, 19) = 3.870, *p =* .064, η_p_^2^ = .169, with the eyes fixating more salient regions in phase 1 compared to phase 2, and no significant main effect of fixation index and group, both *F’*s < 1. There was a significant two-way interaction between painting and phase, *F*(4, 76) = 3.223, *p =* .017, η_p_^2^ = .145, and a significant two-way interaction between phase and fixation index, *F*(4, 76) = 2.801, *p =* .032, η_p_^2^ = .128. There was also a significant three-way interaction between phase, fixation index and group, *F*(4, 76) = 3.037, *p =* .022, η_p_^2^ = .138. No other significant interactions were observed, all *F*’s < 1.20.

**Fig 4:**
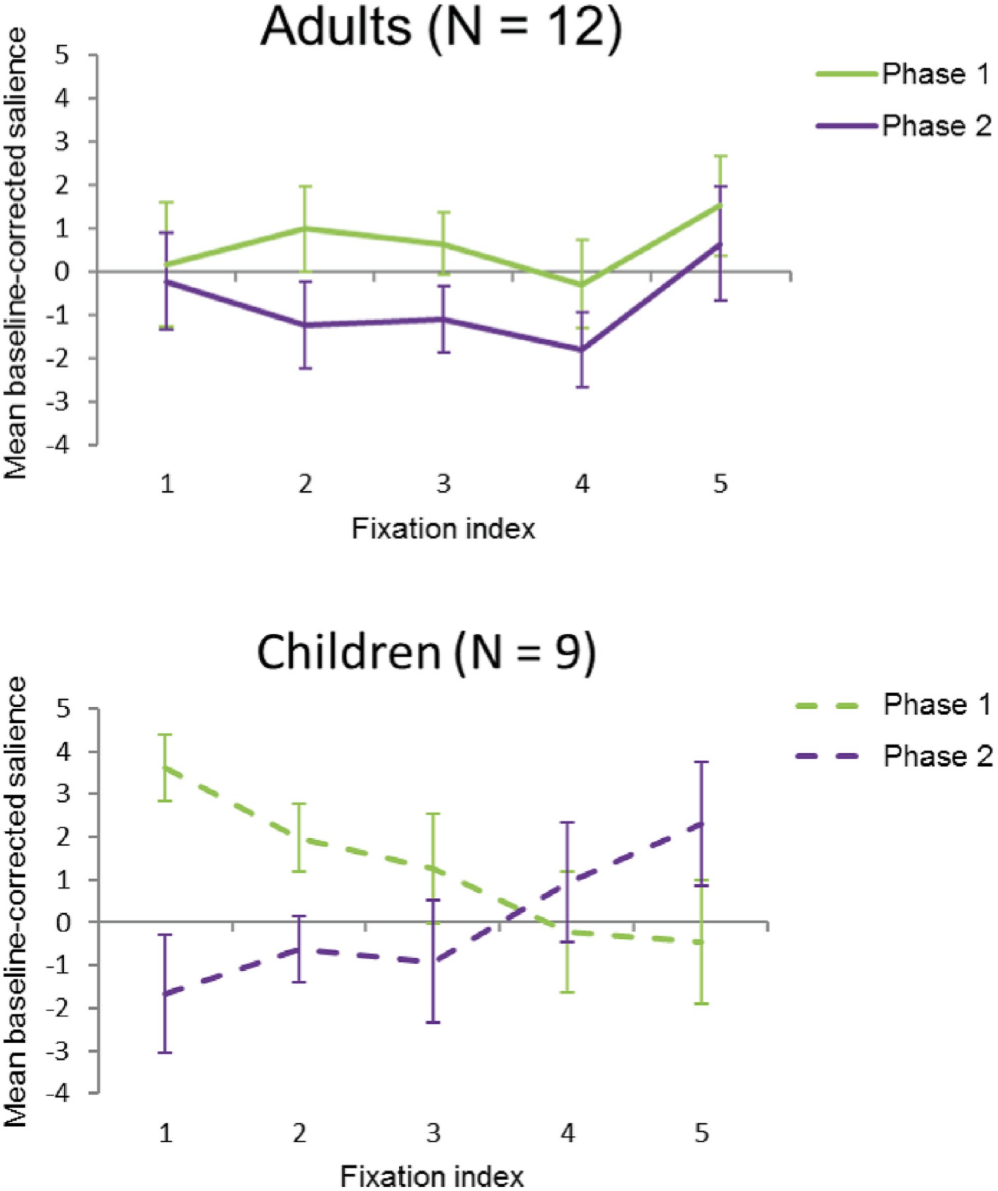
Mean baseline-corrected salience values over fixation index per phase. In both phase 1 and phase 2, the first five fixations of adults (A) land on regions with similar salience values, whereas for children (B), the first fixation in phase 1 lands on high-salient regions, after which following fixations land on regions with gradually declining salience values; vice versa, the first fixation in phase 2 lands on regions with a low salience value, and following fixations land on regions with an increasing salience value. CC BY license, with permission from F. Walker, VU Amsterdam. Error bars in this and following figures represent standard error of the means.

Given the different sizes of the paintings, between painting differences were not investigated further. To further investigate the three-way interaction between phase, fixation index and group, we calculated the slopes of the linear regression line through the individual data points of baseline-corrected salience at fixation over fixation index and the mean baseline-corrected salience over the first five fixations together. For adults, comparing the slopes between phase 1 (mean = 0.15) and phase 2 (mean = 0.12), showed no significant difference in the pattern of salience over fixation index, *t*(11) < 1, *d* = 0.017. Furthermore, although Fig 4 might seem to confirm the trending main effect of phase, the mean baseline corrected salience of the first five fixations together did not differ significantly between phase 1 (mean = 0.601) and phase 2 (mean = -0.7497), *t*(11) = 1.596, *SE* = .846, *p* = .139, *d* = 0.461. This suggests that adults were driven by salience to an equal extent for their first five fixations within each phase, but also that overall they were driven by salience to an equal extent during phase 1 and phase 2. For children, comparing the slopes between phase 1 (mean = -1.032) and phase 2 (mean = 0.948), showed that there was a significant difference in the pattern of salience over fixation index, *t*(8) = 4.596, *SE* = .431, *p* = .002, *d* = -1.532. This suggests that in phase 1, the early fixations of children tend to fall on more salient regions of the painting, with salience at fixation progressively declining over the first 5 fixations. In phase 2, the opposite pattern is found, with fixations progressively landing on more salient regions. Furthermore, the mean baseline corrected salience of the first five fixations together did not differ significantly between phase 1 (mean = 1.234) and phase 2 (mean = 0.008), *t*(8) = 1.216, *SE* = 1.008, *p* = .259, *d* = 0.405, for children. This indicates that the salience pattern of the first five fixations on the paintings is different for children in phase 1 compared to phase 2, with the eyes linearly fixating from more to less salient regions in phase 1 and from less to more salient regions in phase 2. However, although the patterns differ, we observed that children fixated equally salient regions in phase 1 compared to phase 2 when comparing the first five fixations together. This shows that salience effects are most likely present only in some of the five analyzed fixations. Indeed, as Fig 4 already suggests, comparing the individual fixation indices between phase 1 and phase 2 showed that salience at the first fixation, *t*(8) = 2.806, *SE* = 1.878, *p* = .023, *d* = 0.936, and second fixation, *t*(8) = 2.552, *SE* = 1.015, *p* = .034, *d* = 0.851, differed significantly between phase 1 and 2, but that fixation three, four and five did not, all *t*(8)’s < 1.473. This indicates that the first two fixations that children made on the paintings were significantly more driven by salience in phase 1 compared to phase 2. Altogether, the three-way interaction between phase, fixation index and group suggests that the first five fixations of adults, in both phase 1 and phase 2, tend to land on regions with similar salience values, whereas for children, the first fixation in phase 1 tends to land on high-salient regions, after which following fixations tend to land on regions with gradually declining salience values; conversely, the first fixation in phase 2 tends to land on regions with a low salience value, and following fixations tend to land on regions with an increasing salience value (see Fig 4).

### Regions of Interest

From all eye movements, we calculated the absolute time and the percentage of time spent in ROI’s (see Table 3 and Table 4).

**Table 3:** Adults’ fixation time spent in ROI’s as a percentage of total fixation time spent on painting surface (Time in ROI’s) and total fixation time spent on painting surface (Total time). Note that the names of the paintings are abbreviated.

Adults (*N* = 12)	Daubigny	Auvers	Farmhouse	Twilight	Tree Roots
Phase1					
Time in ROI’s (%)	28.5%	32.5%	16.0%	7.9%	22.1%
Total time (sec)	20.6	21.7	18.5	20.2	22.9
Phase2					
Time in ROI’s (%)	30.5%	49.4%	20.7%	8.0%	22.8%
Total time (sec)	22.6	22.6	21.9	22.4	21.0

**Table 4:** Children’s fixation time spent in ROI’s as a percentage of total fixation time spent on painting surface (Time in ROI’s) and total fixation time spent on painting surface (Total time). Note that the names of the paintings are abbreviated.

Children (*N* = 9)	Daubigny	Auvers	Farmhouse	Twilight	Tree Roots
Phase1					
Time in ROI’s (%)	25.8%	29.7%	13.5%	6.2%	11.1%
Total time (sec)	16.7	18.5	17.7	16.5	17.7
Phase2					
Time in ROI’s (%)	31.9%	30.6%	15.5%	7.8%	21.4%
Total time (sec)	18.9	17.4	18.3	17.0	17.6

**Fig 5:**
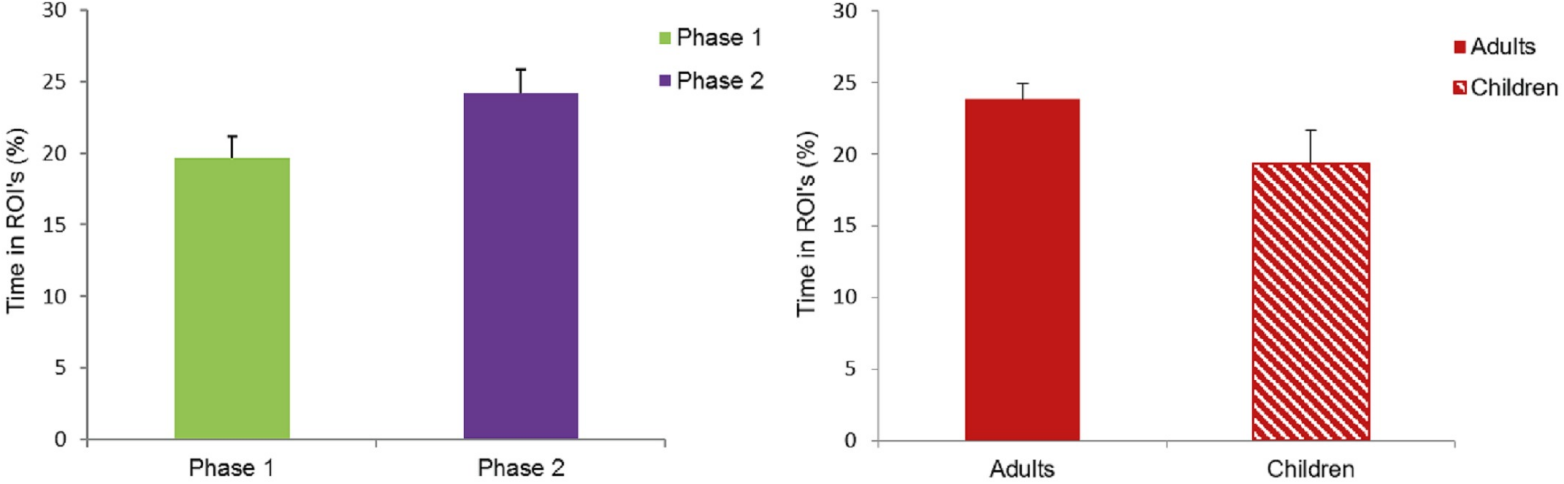
Fixation time spent in ROI’s as a percentage of total fixation time spent on painting surface. A) Participants spent a significantly larger proportion of their fixation time in the ROI’s in phase 2 compared to phase 1. B) Adults spent a slightly larger proportion of their fixation time in the ROI’s compared to children, but the difference was only marginally significant. CC BY license, with permission from F. Walker, VU Amsterdam.

An analysis of variance (ANOVA) on mean fixation time spent in ROI’s with painting (painting 1–5), and phase (phase 1/phase 2) as within-subject factors, and group (children/adults) as between-subjects factor showed a main effect of painting, *F*(4, 176) = 46.412, *p <* .001, η_p_^2^ = .710, a significant main effect of phase, *F*(1, 19) = 4.898, *p =* .039, η_p_^2^ = .205, with more percentage of time spent in ROI’s in phase 2 (mean = 24.2%) compared to phase 1 (mean = 19.6%), and a trending main effect of group, *F*(1, 19) = 3.212, *p =* .089, η_p_^2^ = .145, with a larger percentage of time spent in ROI’s for adults (mean = 23.8%) compared to children (mean = 19.4%). There were no significant two-way interaction effects, all *F*’s < 1.68, and no significant three-way interaction effect, *F*(4, 76) = 2.212, *p =* .103 (Greenhouse-Geisser corrected), η_p_^2^ = .104 (see Fig 5).

**Fig 6:**
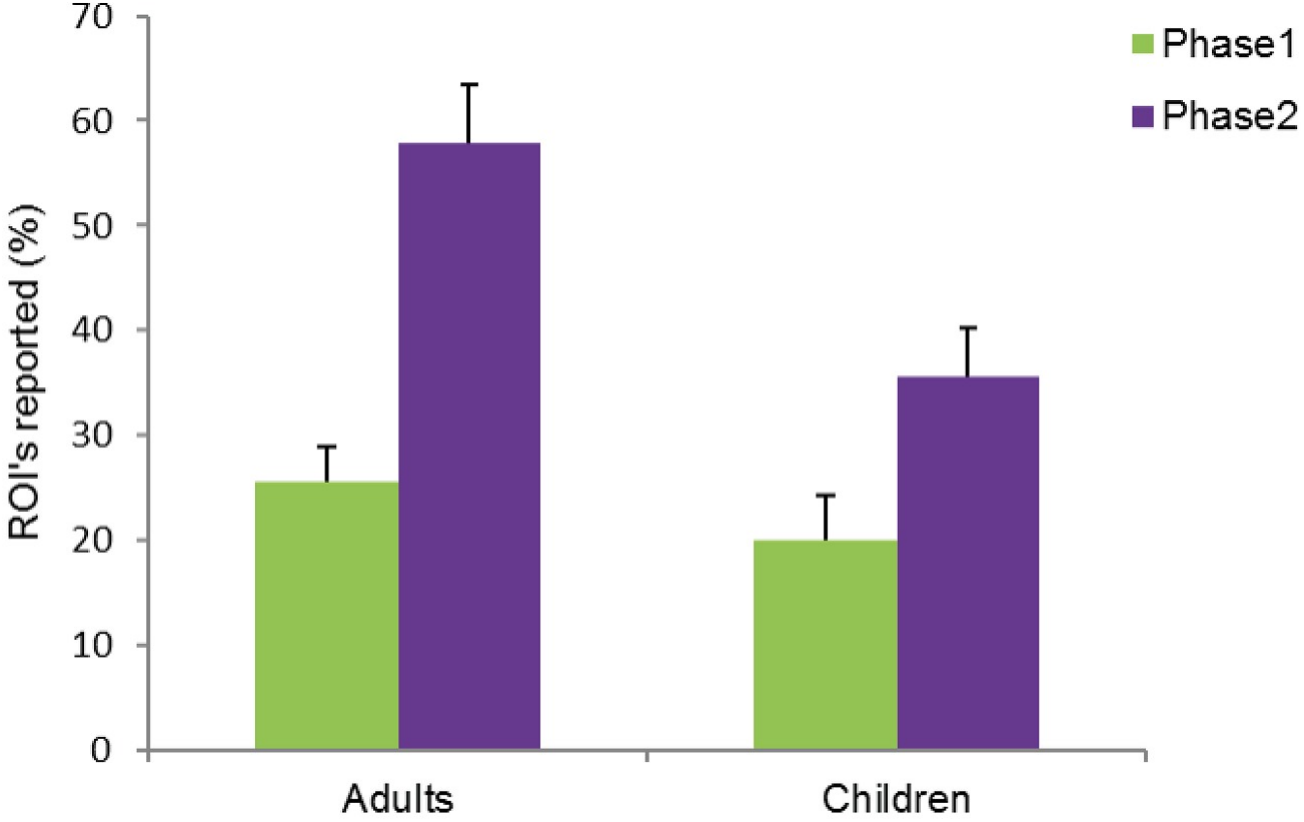
Percentage of ROI’s reported by adults and children in the post phase interviews, presented separately for phase 1 and phase 2. Compared to children, adults reported a significantly greater percentage of ROI’s. A significantly greater percentage of ROI’s was reported in phase 2 compared to phase 1. The interaction between group (adults/children) and post phase interview (phase 1/phase 2) was close to significance. CC BY license, with permission from F. Walker, VU Amsterdam.

We calculated the percentage of ROI’s that were reported by each participant, separately for post phase interview 1 and post phase interview 2. An analysis of variance (ANOVA) on the percentage of ROI’s reported by participants, with painting (painting 1–5), and phase (phase 1/phase 2) as within-subject factors and group (children/adults) as between-subjects factor, showed a significant main effect of painting, *F*(4, 76) = 14.983, *p <* .001, η_p_^2^ = .441, a significant main effect of phase, *F*(1, 19) = 25.199, *p <* .001, η_p_^2^ = .570, with a greater percentage of ROI’s reported in phase 2 (mean = 47%) compared to phase 1 (mean = 23%), and a main effect of group, *F*(1, 19) = 7.424, *p =* .013, η_p_^2^ = .281, with a greater percentage of ROI’s reported by adults (42%) compared to children (28%). A close to significant two-way interaction between phase and group, *F*(1,19) = 3.066, *p* = .096, η_p_^2^ = .139 (see Fig 6) was also observed. Post-hoc paired samples two-tailed t-tests showed a significant increase in the percentage of ROI’s reported by adults in phase 1 (26%) compared to phase 2 (58%), *t*(11) = 5.279, *SE* = 6.104, *p* < .001, *d* = -1.524. Children showed a close to significant difference between the percentage of ROI’s reported in phase 1 (20%) compared to phase 2 (36%), *t*(8) = 2.105, *SE* = 7.391, *p* = .068, *d* = -0.701.These results indicate that both children and adults reported a greater percentage of ROI’s in phase 2 compared to phase 1. However, the interaction that is trending towards significance suggests that the increase in the percentage of ROI’s reported in phase 2 compared to phase 1 was greater for adults compared to children.

**Fig 7:**
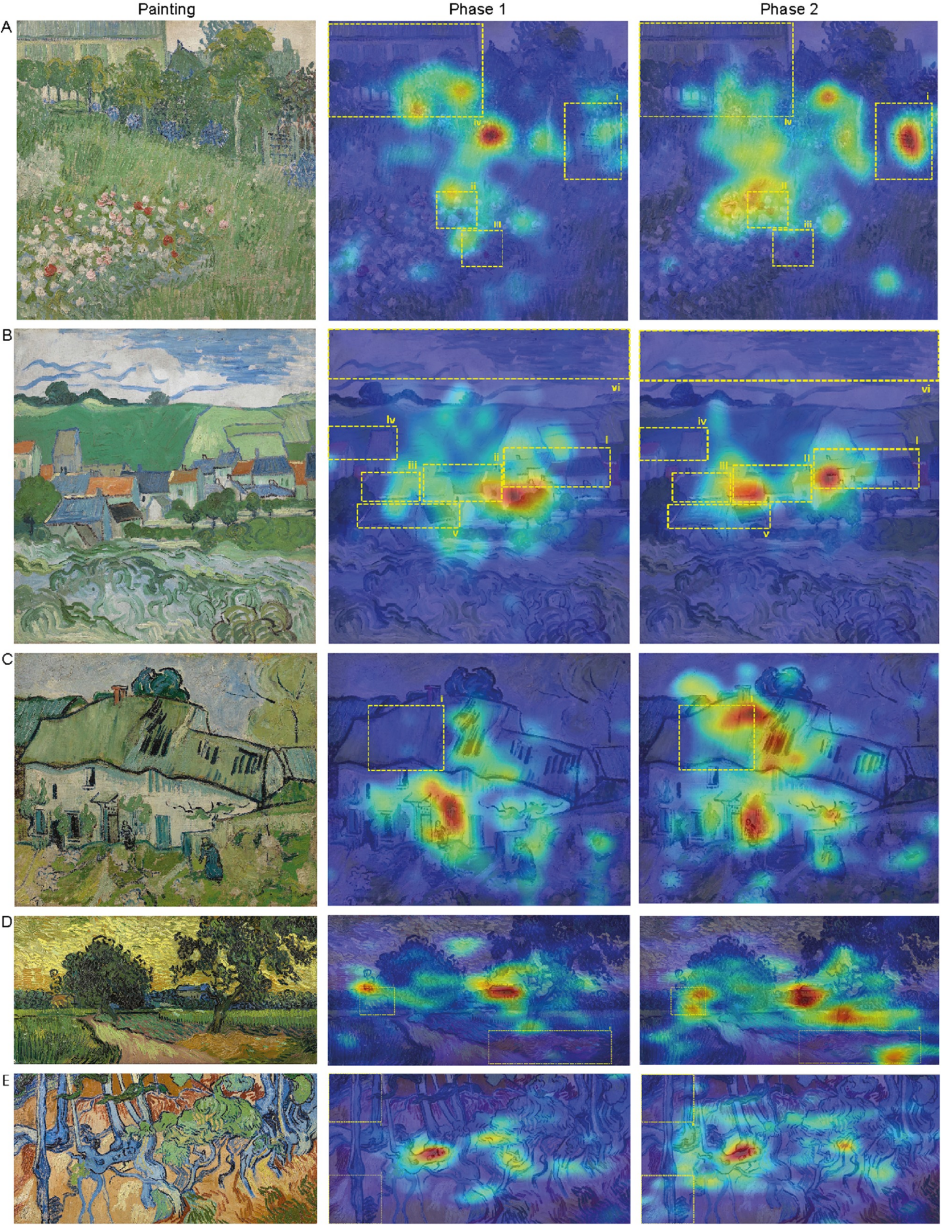
Heat maps adults (ordered as in Fig 3). Adults tend to spend more time in the ROI’s in phase 2 than in phase 1. Painting images downloaded from the official website of the Van Gogh Museum, Amsterdam, under a CC BY license, with permission from J. van Kregten, Van Gogh Museum, Amsterdam (Vincent van Gogh Foundation).

**Fig 8:**
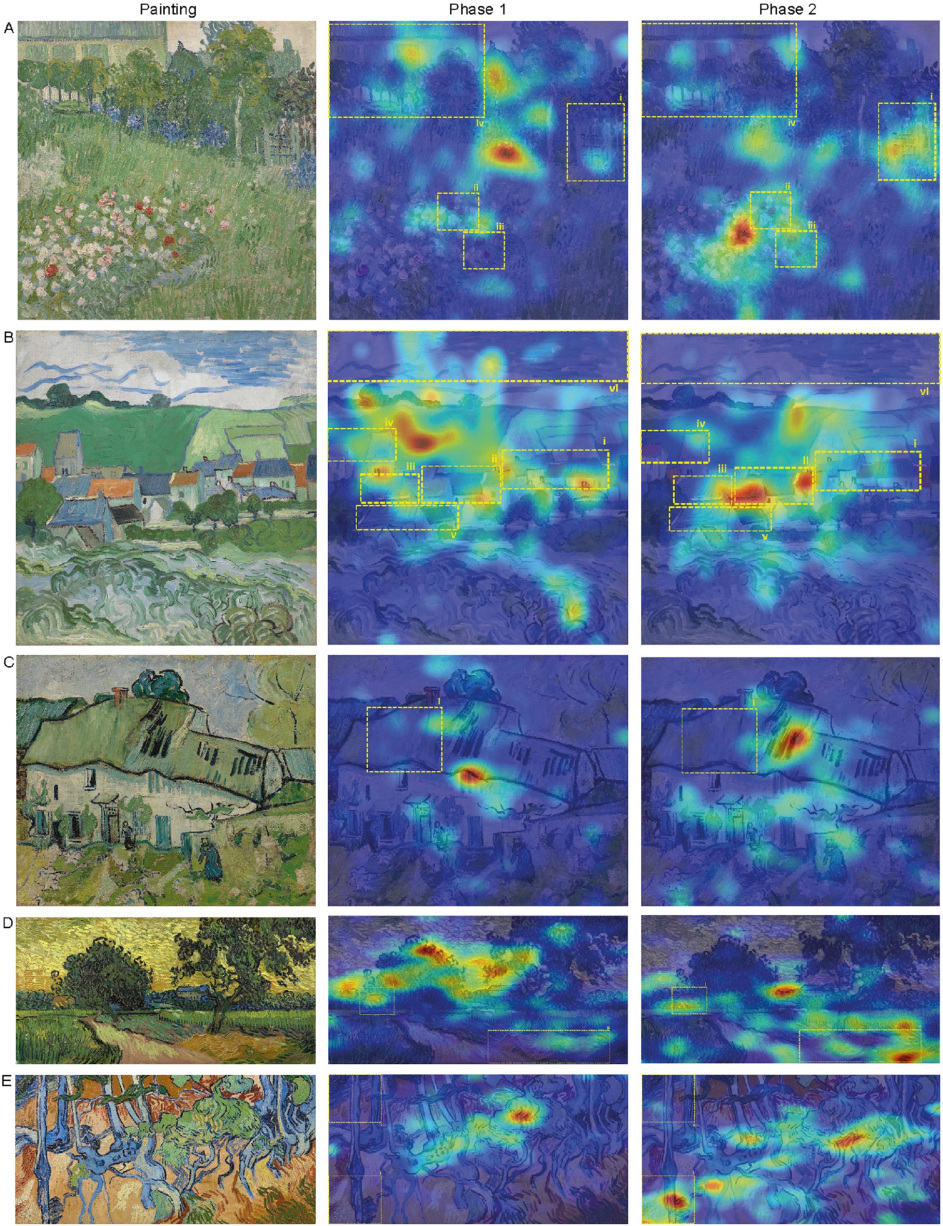
Heat maps children (ordered as in Fig 3). Children tend to spend more time in the ROI’s in phase 2 than in phase 1. Painting images downloaded from the official website of the Van Gogh Museum, Amsterdam, under a CC BY license, with permission from J. van Kregten, Van Gogh Museum, Amsterdam (Vincent van Gogh Foundation).

We created heat maps, to visualize the viewing patterns of participants. The heat maps reflect at what parts of the painting participants fixated, taken the number of fixations and fixation duration into account. Parts that are fixated more often and/or fixated longer are presented in red, scaling down to blue for regions that are fixated less often and/or fixated for shorter times. To interpret the above mentioned results within the context of the heat maps, we created separate heat maps per group (adult/children), phase (phase 1/phase 2) and painting (painting 1–5). Moreover, the outlines (i.e., yellow dashed lines in Fig 7 and Fig 8) of the different ROI’s are plotted as well. The heat maps in Fig 7 and Fig 8 illustrate participants’ tendency to fixate on ROI’s more often and/or for a longer time in phase 2 compared to phase 1.

**Fig 9:**
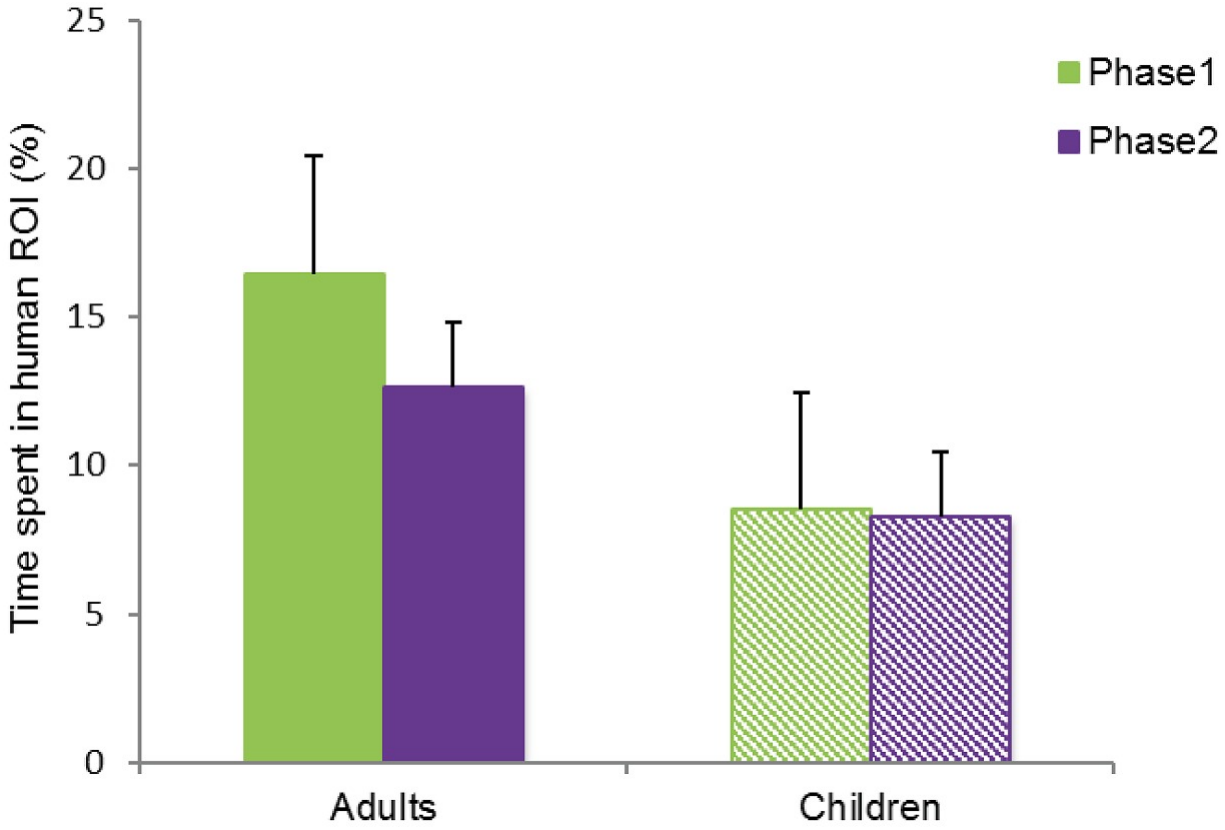
Percentage of time that adults and children spent in ROI "human", painting Tree Roots. In phase 1, adults spent more time than children looking at the “human figure”. CC BY license, with permission from F. Walker, VU Amsterdam.

In the interviews at the end of phase 1, 10 out of 12 adults reported that they had seen a “human figure” in the painting Tree Roots, whereas only 2 out of 9 children reported this. To examine whether adults also spent more time looking at this human-like figure, we calculated the time spent in the ROI “human figure” as a percentage of the total time spent on the painting surface. A one-tailed independent samples t-test confirmed that in phase 1 adults (mean = 16.4%) spent more time looking at the “human figure” than children (mean = 8.5%), *t*(19) = 1.857, *p* = .039, *d* = 1.199 (see Fig 9).

**Fig 10:**
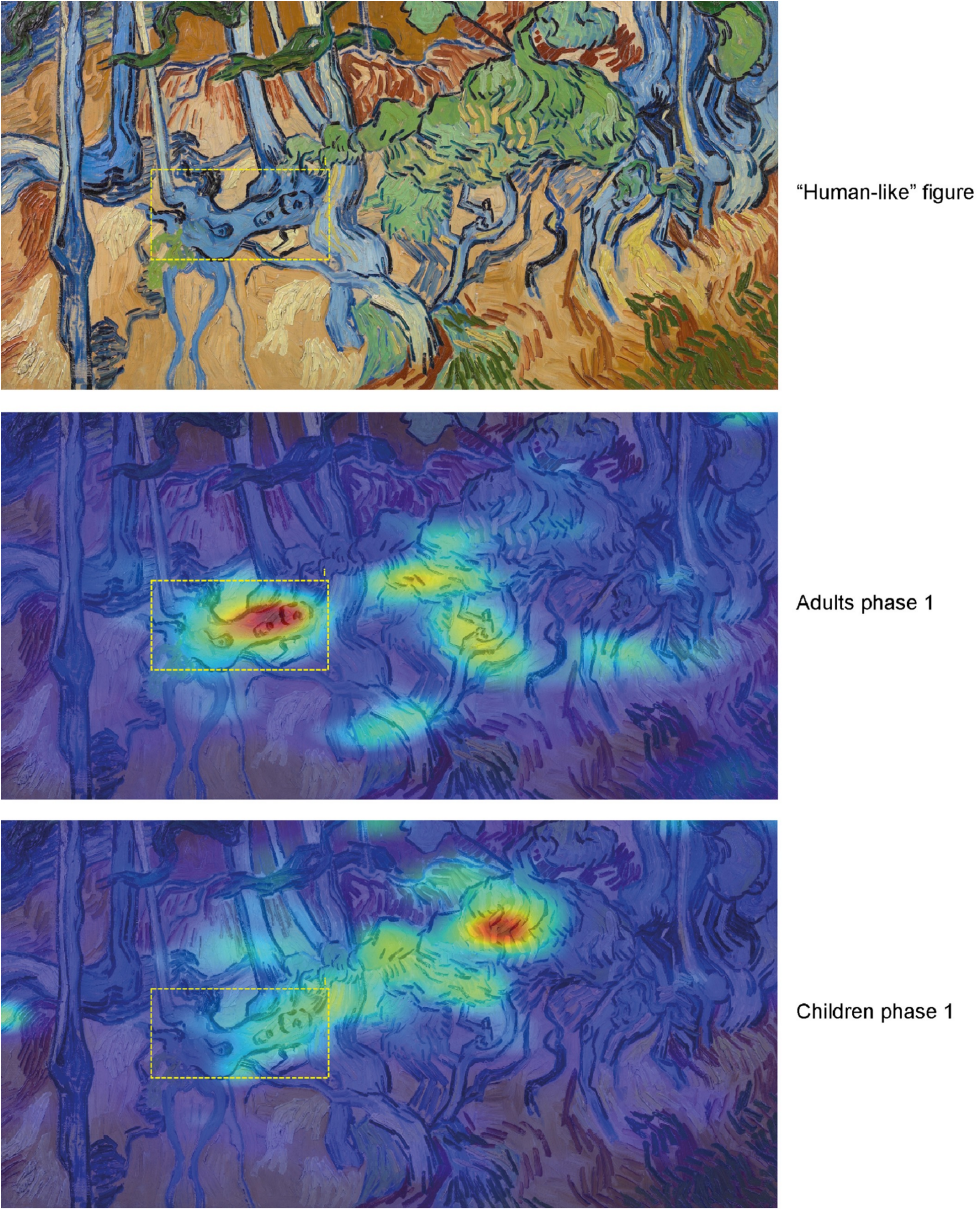
Heat maps for painting "Tree Roots", phase 1. Adults pay attention to the "human-like" figure. Children ignore the figure. Painting image downloaded from the official website of the Van Gogh Museum, Amsterdam, under a CC BY license, with permission from J. van Kregten, Van Gogh Museum, Amsterdam (Vincent van Gogh Foundation).

Heat maps for "Tree Roots" are consistent with these results (see Fig 10).

As the human-like figure was not mentioned in the descriptions read out to the participants before phase 2, we did not expect any differences between adults and children in phase 2. This prediction was confirmed by a two-tailed independent samples t-test comparing the proportion of time spent in the ROI for adults (mean = 13%) and children (mean = 8%), *t*(19) = 1.026, *p* = .318, *d* = 0.651.

## Discussion

In the present study we used “eye movements in natural behaviour” [35] as a way of investigating how children and adults look at actual paintings in a museum setting. Eye movements are an index of overt visual selection, and reflect the outcome of competition between external environmental factors driving eye movements and internal factors such as goals, beliefs and knowledge [9]. Analysis of the observer's eye movements while viewing paintings can help shed light on the respective contribution of bottom-up and top-down processes in the first stages of his/her aesthetic experience.

In phase 1 of our study, participants were instructed to freely view each of the five paintings for 30 seconds, to investigate the influence of bottom-up (stimulus driven) processes on eye movement behaviour. Top-down processes were manipulated by providing background information about each painting before the start of phase 2. This information was in line with the information that is typically given to visitors of the Van Gogh Museum, Amsterdam. To further quantify the role of top-down and bottom-up processes we tested adults and primary school children. It is known that children rely more on bottom-up factors than on top-down factors when looking at pictures [36].

Results of the salience analysis show that, in phase 1, children initially focused on the salient regions of the paintings, later shifting their eye gaze to less salient regions—exactly the pattern predicted by low level salience models (e.g., [14, 15]). Conversely, in phase 2, after they received background information about the paintings, they fixated less salient regions first, only later shifting to more salient regions. This suggests that, in phase 2, children’s eye movements were more influenced by top-down factors. Taken together, these findings indicate that children’s eye movement behaviour is initially driven by low-level, bottom-up factors. After receiving background information about the painting, the eye movement behaviour drastically changes: Now areas with low salience values are inspected first. This implies that, in phase 2, top-down knowledge (the background information) drives the eye movement pattern much more than low level salience features.

In contrast, the eye movement behaviour of the adults show relatively little change between phase 1 and 2. This result indicates that low level salience did not play much of a role in guiding adult eye movement patterns. In both phase 1 and 2, in fact, adults fixated areas with low salience values. This result is inconsistent with strict low level salience models of eye movement control (e.g., [14, 15]) and suggests that adults’ eye movements in phase 1 were already influenced by top-down factors, such as knowledge and the gist of the scene conveyed by the painting. Overall, these results match previous findings, showing that children rely more on bottom-up guidance than adults [36] and that adult observers focus their gaze on specific areas of a painting [20]. Crucially, our results also show than children use top-down factors more when prompted to do so by the provision of suitable background information.

On the basis of the information provided about the paintings, we defined those areas (ROI’s) that were directly or indirectly mentioned in the painting description. Following these descriptions, viewers spent significantly more time in the regions that were mentioned, compared to when they did not have this information. Since adults spent more time in the Regions of Interest than children, it seems that the manipulation had a larger effect on them than on the children. Participants’ verbal reports were consistent with their observed fixation behaviour. Adults had a general tendency to mention important regions in the paintings; after they had received background information about the paintings, this tendency became even stronger. Overall, the aforementioned findings are in line with our salience results and show that, by playing a causal role in determining eye movement behaviour, background information can alter the way a visitor views a painting on display at a museum–a result of great value for museum educators.

One unexpected finding was that 10 out of 12 adults but only 2 out of 9 children reported the presence of a “human figure” in Tree Roots, a feature that was not mentioned in the background information. Again the self-reports corresponded to the eye movement data, showing that adults spent more time looking at this particular region compared to children. This finding is consistent with the notion that people tend to see people, faces or animals in naturally occurring patterns, a phenomenon known as pareidolia. Other authors have suggested that the human brain is wired to detect bodies and faces even if we are not looking for them (e.g., [46, 47]). In the present experiment, pareidolia was mainly observed in adults, suggesting that the phenomenon is mainly driven by top-down factors, such as expectations or experience.

Our study has some limitations that should be acknowledged. Participants increased attention to ROI’s in phase 2 relative to phase 1 could have been influenced (but not determined) by the repeated exposure to the same paintings. Even though this is a potentially valid criticism, it is important to note that the information provided to the participants after phase 1 strongly affected the eye movement pattern seen in phase 2. If only the second exposure to the paintings was driving this effect (and not the information provided) we would not have found such a strong relationship between the information provided and the subsequent eye movement pattern. A second issue is the relatively small sample size (21 participants). Even though our sample is quite substantial for this type of real life experiments, and larger than those seen in the majority of previous studies, a larger sample would obviously have been preferable. Unfortunately, it was not possible to accommodate a larger sample within the security and time constraints of a study conducted in a working museum during public opening hours.

Despite the minimalistic design of the mobile eye tracker, it was not possible to hide the equipment from other visitors, so it is theoretically possible the presence of camera-wearing participants may have affected visitor behaviour, and that this may have had a feedback effect on participants. Nevertheless, we saw no evidence for such effects: None of our participants reported they had been bothered by the device (during and after the experiment), or by the reactions of other visitors.

Given the limitations of our study, it is clear that much research remains to be done. We believe, nonetheless, that The Van Gogh Museum Eye-tracking Project represents an important step forward. What we have shown is that basic aspects of art perception, such as top-down and bottom-up guidance of eye movements, can indeed be studied in a real museum setting and that such studies can yield valuable results: In our case, the discovery of potentially important differences in the way children and adults perceive art under ecologically valid conditions.

## Appendix–Painting descriptions

### English descriptions

#### Daubigny's Garden

Van Gogh painted the garden of Daubigny, a painter he admired. Daubigny’s house is in the background. On the right you can see the gate that led to the neighbours’ garden. Van Gogh wanted to have two main colours here: pinkish-red and green. These colours make each other stronger. Because he was out of canvas, he used a tea towel. He first applied a ground layer to the towel. Once that was dry, he could paint over it. The ground layer was once bright pink. You could see it clearly between the blades of grass in the foreground. Unfortunately, almost all the pinks and reds have partly or completely faded over the years. Only a few roses are still bright red. Van Gogh used a different type of red paint for these flowers, which did not change colour.

#### View of Auvers

This landscape is a painted sketch, which Van Gogh dashed off quickly. He did not pay equal attention to all parts of it and even decided not to finish painting the sky. We know that Van Gogh did not use grey. Even so, we see the colour grey in some of the housetops. They probably used to be red, but the colour of the paint has changed over the years, and red has faded to grey.

#### Farmhouse

Van Gogh painted this broken-down farmhouse with more than just brushes. For the roof, he used a palette knife. That is clear if you look to the left of the chimney, where the paint has been spread out in a smooth, attractive way. Van Gogh painted the foreground quickly, using more paint in some places than in others. You can see bits of bare canvas between the brushstrokes.

#### Landscape at Twilight

Here, Van Gogh painted trees that stand out darkly against the vivid colour of the evening sky. The sun has just set, and the house on the left catches the last rays of sunlight. Van Gogh used many different types of brushwork. For example, he used small, round strokes for the leaves of the trees and short, straight dashes for the wheat. But there is also paint on this painting that does not belong there. The specks of green paint on the hay in the right foreground come from a different painting. Van Gogh occasionally stacked up his painted canvases, and sometimes they were not yet completely dry. That explains why some paint came off the painting that was stacked against this one.

#### Tree Roots

Only after looking at this painting for a while do you realise that it shows tree trunks and roots. The trees are on the edge of a mound dug out of a marl pit. A sliver of blue sky is visible at the upper left. Because the trees are regularly chopped down, you see many gnarled stumps. The edge of the mound has crumbled away and the trees are almost falling off. Their roots are already exposed. This is Van Gogh’s last painting. He never completed it. That is clear if you look at the lower left corner. He had not yet painted contour lines around the blue roots there, as he did around the ones on the right. You can also see that there is not as much paint in the lower left corner of the canvas as there is in the upper part. If you look at the tree trunk on the far left, you can even see bits of bare canvas between the brushstrokes.

### Dutch descriptions

#### Daubigny's Garden

Van Gogh schilderde de tuin van Daubigny, een kunstenaar die hij bewonderde. Achteraan staat Daubigny’s huis. Rechts zie je het hekje waardoor je naar de tuin van de buren kon lopen. Van Gogh wilde hier 2 kleuren belangrijk maken: roze-rood en groen. Die kleuren maken elkaar sterker. Omdat zijn schilderdoek op was, pakte hij een theedoek. Daar smeerde hij eerst een grondlaag op. Toen die droog was, kon hij erop schilderen. De grondlaag was vroeger fel roze. Tussen de grassprieten vooraan kon je die laag goed zien. Helaas is bijna al het roze en rood in de loop der tijd verbleekt of verdwenen. Alleen een paar roosjes zijn felrood gebleven. Van Gogh gebruikte voor deze bloemen andere rode verf, die niet verkleurde.

#### View of Auvers

Dit landschap is een schets in verf. Van Gogh maakte het snel en vlot. Hij gaf niet alles evenveel aandacht. De lucht maakte hij zelfs expres niet af. We weten dat Van Gogh geen grijs gebruikte. Toch hebben sommige daken van de huizen die kleur. Waarschijnlijk waren ze ooit rood. In de loop der jaren verkleurde de verf. Het rood is verbleekt tot grijs.

#### Farmhouse

Deze vervallen boerderij heeft Van Gogh niet alleen met penselen geschilderd. Voor het dak gebruikte hij een paletmes. Dat zie je links van de schoorsteen: de verf is daar mooi glad uitgesmeerd. De voorgrond heeft hij snel geschilderd. Niet overal zit evenveel verf: je ziet het kale doek tussen de verfstreken door.

#### Landscape at Twilight

Van Gogh schilderde hier bomen die donker afsteken tegen een gekleurde avondlucht. De zon gaat net onder. Op het huisje links zie je de laatste zonnestralen. Van Gogh gebruikte veel verschillende verfstreken. Zoals ronde haaltjes voor de bladeren van de bomen, en korte rechte streepjes voor het koren. Maar er zit ook verf op het schilderij die er niet hoort. De kleine groene vlekjes op het hooi in de voorgrond rechts komen van een ander schilderij. Van Gogh stapelde soms zijn doeken op elkaar. Ze waren dan niet altijd helemaal droog. Het schilderij dat tegen dit werk aanlag, heeft dus verf afgegeven.

#### Tree Roots

Pas als je langer naar dit schilderij kijkt, ontdek je dat het boomstammen en -wortels zijn. De bomen staan op de rand van een uitgehakte heuvel van een mergelgroeve. Linksboven is nog een stukje blauwe lucht te zien. Omdat mensen de bomen regelmatig omhakten, zie je veel knoestige stronken. De rand is afgebrokkeld en de bomen vallen er bijna af: hun wortels liggen al bloot. Dit is Van Gogh’s laatste schilderij. Het was niet af. Dat zie je linksonder: de blauwe wortels hebben nog geen omtreklijntjes zoals de wortels rechts. Ook zit er linksonder niet zoveel verf op het doek als bovenaan. In boomstronk helemaal links zie je zelfs het kale doek tussen de verfstreken door.
